# Human Hippocampus Arbitrates Approach-Avoidance Conflict

**DOI:** 10.1016/j.cub.2014.01.046

**Published:** 2014-03-03

**Authors:** Dominik R. Bach, Marc Guitart-Masip, Pau A. Packard, Júlia Miró, Mercè Falip, Lluís Fuentemilla, Raymond J. Dolan

**Affiliations:** 1 Wellcome Trust Centre for Neuroimaging, University College London, London WC1N 3BG, UK; 2 Berlin School of Mind and Brain, Humboldt University Berlin, 10099 Berlin, Germany; 3 Psychiatric Hospital, University of Zurich, 8032 Zurich, Switzerland; 4 Age Research Center, Karolinska Institute, 17111 Stockholm, Sweden; 5 Cognition and Brain Plasticity Unit, Institute of Biomedicine Research of Bellvitge (IDIBELL), 08908 Barcelona, Spain; 6 Epilepsy Unit, University Hospital of Bellvitge, 08907 Barcelona, Spain; 7 Department of Basic Psychology, University of Barcelona, 08007 Barcelona, Spain

## Abstract

Animal models of human anxiety often invoke a conflict between approach and avoidance [1, 2]. In these, a key behavioral assay comprises passive avoidance of potential threat and inhibition, both thought to be controlled by ventral hippocampus [2–6]. Efforts to translate these approaches to clinical contexts [7, 8] are hampered by the fact that it is not known whether humans manifest analogous approach-avoidance dispositions and, if so, whether they share a homologous neurobiological substrate [9]. Here, we developed a paradigm to investigate the role of human hippocampus in arbitrating an approach-avoidance conflict under varying levels of potential threat. Across four experiments, subjects showed analogous behavior by adapting both passive avoidance behavior and behavioral inhibition to threat level. Using functional magnetic resonance imaging (fMRI), we observe that threat level engages the anterior hippocampus, the human homolog of rodent ventral hippocampus [10]. Testing patients with selective hippocampal lesions, we demonstrate a causal role for the hippocampus with patients showing reduced passive avoidance behavior and inhibition across all threat levels. Our data provide the first human assay for approach-avoidance conflict akin to that of animal anxiety models. The findings bridge rodent and human research on passive avoidance and behavioral inhibition and furnish a framework for addressing the neuronal underpinnings of human anxiety disorders, where our data indicate a major role for the hippocampus.

## Highlights

• Human behavior in a spatial approach-avoidance conflict resembles animal behavior
• Threat level in this context specifically engages anterior hippocampus
• Hippocampal lesions reduce passive avoidance and inhibition
• The study provides human evidence for an anterior hippocampus role in anxiety

## Teaser

Using a combined functional magnetic resonance imaging and hippocampus lesion approach, Bach et al. demonstrate that the human anterior hippocampus is engaged in monitoring threat level during approach-avoidance conflict, replicating and extending a rodent literature on models of anxiety.

## Results and Discussion

**Figure 1:**
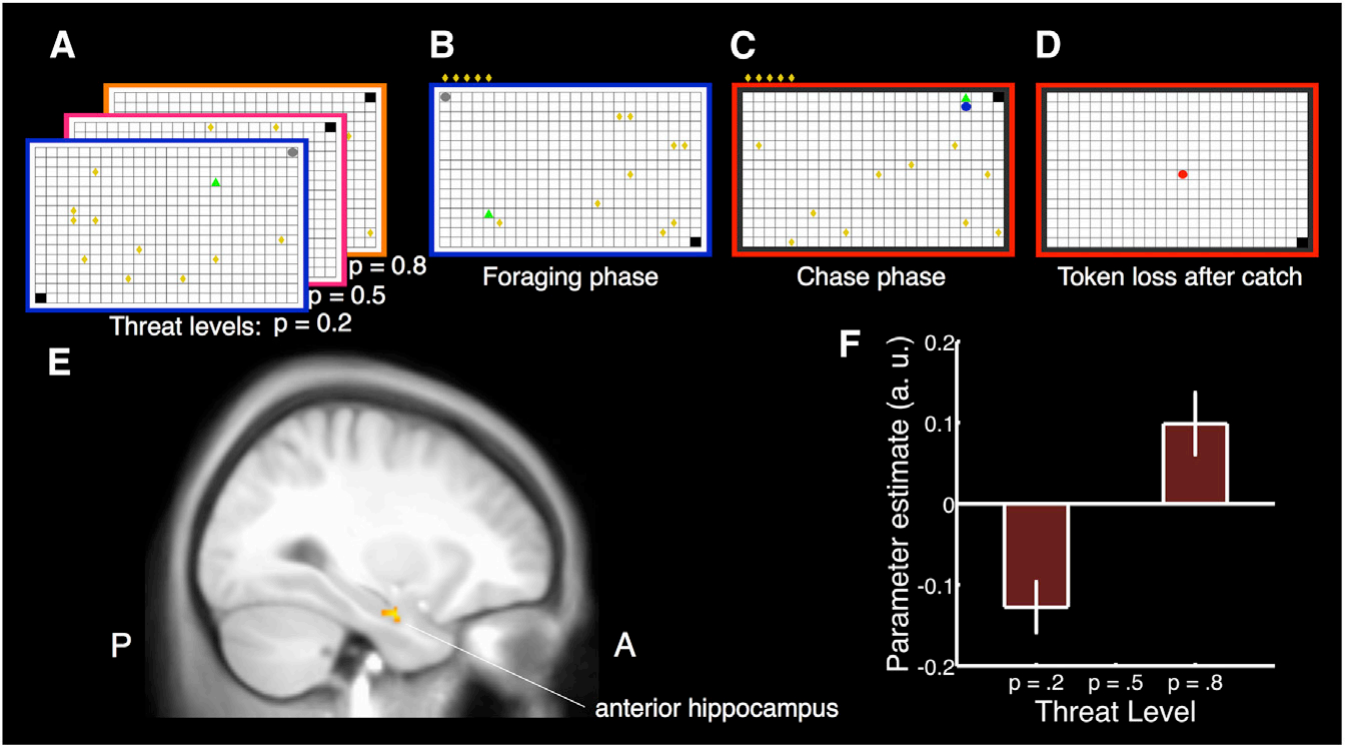
Human Approach-Avoidance Computer Game and Hemodynamic Responses to Threat Level (A) The human player (green triangle) forages for tokens (yellow rhombi) on a 24 × 16 grid. One of three differently dangerous predators (threat levels denoted by different frame colors) looms in a corner of the grid (gray circle). (B) Collected tokens appear on the grid and are paid out for money at the end of the game. (C) The predator can wake up any time and chase the human player. The human player can hide in the safe place (black grid block). (D) If caught, the human player looses all tokens from this epoch, and the epoch is over. (A–D) The possibility of a chase phase (C and D) provides avoidance motivation during the foraging phase (A and B). We report behavior during the foraging phase. (E) Blood-oxygen-level-dependent (BOLD) responses to threat level: activation in the left anterior hippocampus, the human homolog of the rodent ventral hippocampus (cluster peak T = 4.95 at −27/−6/−24 mm MNI, 74 voxels, p < 0.05 small-volume corrected for family-wise error across the region of interest, overlaid on group average T1-weighted image in MNI space, x = −29 mm). See Figure S1 for additional whole-brain analysis. (F) Estimated BOLD activity in the hippocampus cluster for the three threat levels, individually adjusted for BOLD activity at medium threat level. Error bars indicate the SEM difference between low and medium or high and medium threat level. See also Figure S1 and Table S1.

We developed a human approach-avoidance task that emulates rodent anxiety paradigms such as operant conflict tests [11] (e. g. Geller-Seifter test), the elevated plus maze (EPM), and the open field test (OT) and thus differs from paradigms that elicit fear (when threat is acute) [1, 12] or panic (when threat is immiment) [1, 13]. In this task, presented over successive epochs (Figures 1A–1D) in the form of a computer game, collection of monetary tokens on a grid provides approach motivation. The possibility that a virtual predator might wake up and remove all tokens harvested during the epoch provides a potential threat, and thus avoidance motivation. Different from animal tasks that do not involve actual threat (such as EPM or OT), in our task both approach and avoidance motivation were constant over successive trials, so as to avoid habituation. In each epoch, one of three predators representing different levels of threat (corresponding either to chase speed or wake-up probability, in different versions of the task) was present but inactive in a corner of the grid. Participants successfully learned to differentiate the three threat levels (Table S1 available online). The grid corner opposite to that of the predator represented a safe place where the predator could not catch the participant. Participants started either in the same corner as the predator (“active” epoch) or from the safe place (“passive” epoch). From a defensive perspective, active epochs engage active avoidance to escape the predator position initially and passive avoidance later on; passive epochs involve passive avoidance behavior alone [1, 14]. In this report, we focus on the foraging phase of the task, and this corresponds to the time within an epoch where the predator represents a potential threat but is inactive.

We report seven measures that index passive avoidance and behavioral inhibition, inspired by (or analogous to) measures typically used in animal models of anxiety. In particular, we report distance from threat as a summary measure of passive avoidance. We modeled distance from nearest wall as a proxy for center aversion in the OT [3], presence in the threat quadrant as a proxy for open-arm entries in the EPM, presence in safe quadrant as a proxy for center time in the EPM, presence in safe place as a proxy for closed arm time in the EPM [11], and speed when outside the safe place and rate of token collection as summary measure of behavioral inhibition. Respective measures of rodent behavior are interrelated [15], but with a multidimensional structure [11]. We did not analyze specific movement patterns, such as cyclic round trips, as these are difficult to distinguish from foraging-related behaviors in our task, or analogs of postural patterns such as defensive quiescence [16] for which the virtual computer game lacks specificity. Since participants accumulate tokens as the epoch progresses, thus increasing their potential loss, we analyze all measures with respect to intraepoch time.

**Figure 2:**
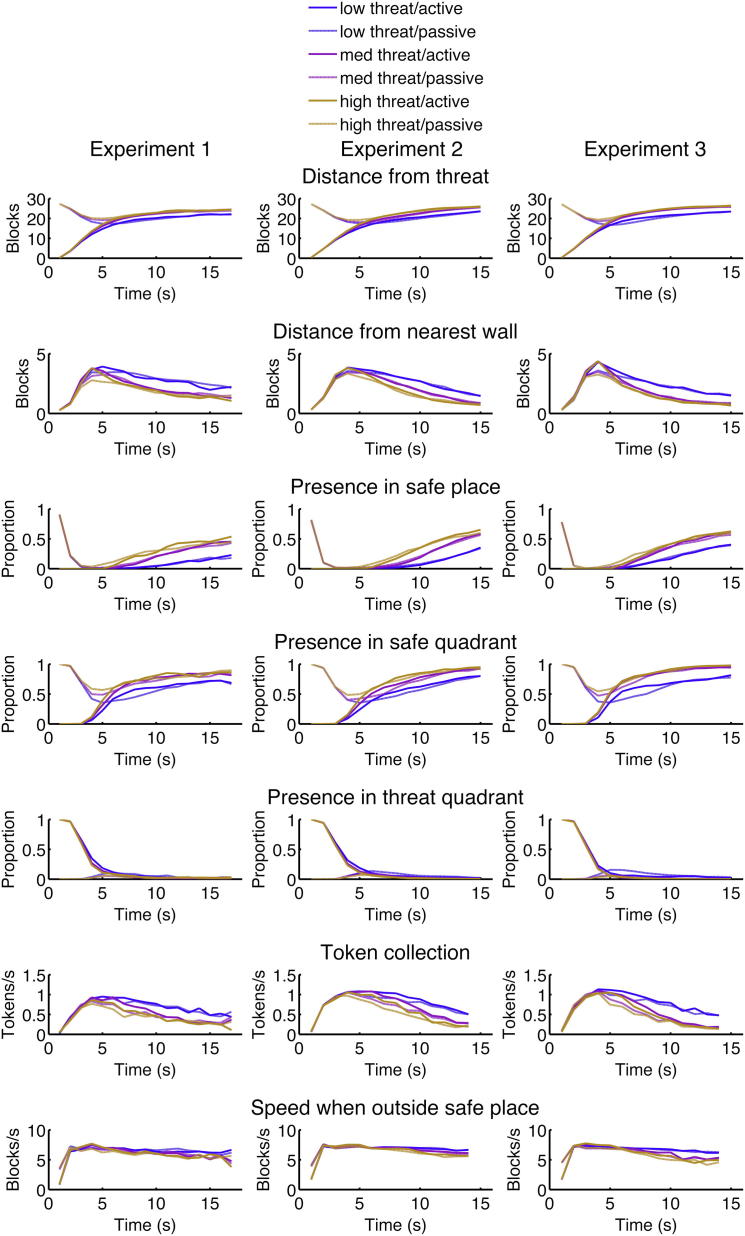
Defensive Behavior in Healthy Humans Data are shown for three experiments (left, middle, and right columns), three threat levels (blue, purple, and orange), and two starting situations (active: start with predator, solid; passive: start opposite predator, dashed). These measures show that behavioral adaptation is consistent across experiments. Inference statistics are given in Table 1. Behavior is adapted according to threat level and intraepoch time. Behavior under the two higher threat levels (purple and orange lines) is more similar to each other than to the lower threat level (blue line), reflected in a quadratic effect of threat level and suggesting a strategy that is not maximizing reward. Naturally, behavior depends on starting conditions (with or opposite of threat) initially, but converges over time.

We conducted three independent experiments involving 24, 25, and 19 healthy volunteers respectively, varying preassessment training (no training, 600 epochs, 480 epochs), epoch duration, and implementation of threat level (experiment 1, chase speed; experiments 2 and 3, wake-up probability). Participants adjusted their behavior according to threat level in both active and passive epochs. For each experiment, we computed a threat level × task (active/passive) × time repeated-measures ANOVA with Greenhouse-Geisser correction for nonsphericity and Bonferroni correction for seven measures per experiment. All behavioral measures adapted to threat level, and this became more pronounced as intraepoch time progressed (linear effect of threat level, and linear-linear interaction of threat level by time; Figure 2, Table 1). In many measures, participants barely discriminated between a medium and high threat situation as time passed (quadratic-linear threat level × time interaction). We note that most of the behavioral adapations that linearly relate to threat level might map to optimal reward-maximizing strategies predicted by standard economic theory. Yet, normative expected utility maximization does not predict nonlinear influences of threat beliefs [17].

Next, using functional magnetic resonance imaging (fMRI), we assessed hippocampal involvement in this behavioral adaptation (experiment 3). We hypothesized that increasing threat level would be associated with an increase in anterior hippocampal blood-oxygen-level-dependent (BOLD) response, the homolog of the rodent ventral hippocampus. We first defined a region of interest (ROI) comprising the bilateral hippocampus using a standard brain atlas. To ensure that observed BOLD responses were not due to spatial navigation or passage of time, we partialled out spatial and time variables on a moment-by-moment basis (position, speed, change of movement direction, distance from predator, token collection, and intraepoch time). Strikingly, we observed a linear positive effect of threat level on BOLD responses in the left anterior hippocampus (Figures 1E and 1F). In an exploratory approach, we analyzed BOLD responses across the whole field of view, which revealed that the hippocampal cluster extended beyond the ROI (112 voxel in total) to encompass 20 voxels in the posterior amygdala, though these did not survive whole brain correction for multiple comparison. Additional clusters, surviving whole-brain correction, were observed in the right inferior frontal gyrus/insula, bilateral parahippocampal gyrus, and right fusiform gyrus (Figure S1). We also performed further analysis of the ROI signal on an epoch-by-epoch basis and found no association of the ROI signal with epoch-summary measures of spatial behavior (relative duration of presence in safe place, average distance from threat, and distance traveled; see the Supplemental Experimental Procecures), only with threat level. This is consistent with the idea that when human participants face an approach-avoidance conflict, the anterior hippocampus is involved in monitoring threat level. Exploratory analysis of between-subject differences revealed that mean ROI signal per subject was related to the mean debriefing estimates of threat probability (p < 0.005; see the Supplemental Experimental Procedures), i.e., subjects with a higher estimate of threat level had higher BOLD signal in this area. This provides additional support for an interpretation that hippocampal activation is related to monitoring of threat level in our task.

**Figure 3:**
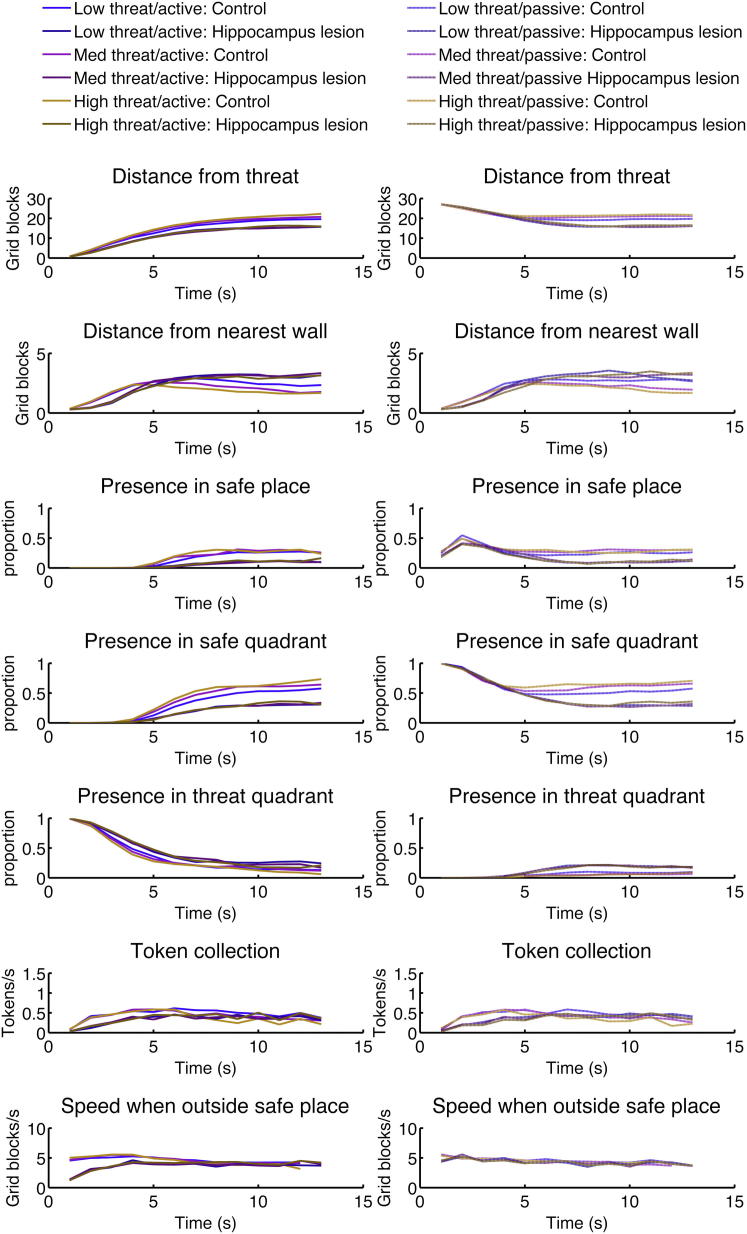
Defensive Behavior in Patients with Hippocampus Lesions Behavioral measures of seven patients with TLE+HS (shaded lines) and 12 age- and gender-matched healthy control participants (solid lines). Left: active, starting with the predator. Right: passive, starting in the safe place. Patients with TLE+HS show reduced anxiety behavior overall than healthy individuals, and this group difference increased with intraepoch time. Further, they show reduced adaption to threat level compared to healthy individuals. See also Figure S2 and Tables S2–S4.

However, fMRI alone does not enable us to establish a causal role for the hippocampus in approach-avoidance behavior. To address this, we investigated seven patients (experiment 4) with temporal lobe epilepsy and selective hippocampal sclerosis (TLE+HS) and an age- and gender-matched group of 12 healthy volunteers (Tables S3 and S4 and Figure S2). In contrast to controls, TLE+HS patients were much less influenced by threat (Figure 3 and Tables 1 and S2). In terms of specific parameters, they showed less presence in the safe place and safe quadrant (effect of group). Across all measures, patients behaved increasingly less cautious than healthy participants as intraepoch time passed (interaction group × time). Across a number of measures, this was more pronounced at higher threat levels (interaction group × threat × time).

To outrule a possibility these findings were secondary deficits, we conducted a number of supporting analyses (see the Supplemental Experimental Procedures). Spatial navigation deficits [18] (or hyperactivity as observed after dorsal rodent hippocampus lesions [3]) were not evident in the patients as indexed by either foraging speed or rate of token collection. A possible spatial deficit is hinted at by the fact that patients moved around with longer strokes, making fewer turns per time unit than healthy controls (group contrast in a full ANOVA model without Bonferroni correction: t = −2.66, p < 0.05). Nevertheless, even if we account for turns per second or for speed, this did not change the pattern of group differences. Likewise explicit knowledge of threat, assessed by post hoc estimates of threat level, did not differ between patients and controls overall which means that this variable cannot account for overall group differences. The interaction of threat level and group approached significance (Table S1; p = 0.06). However, accounting for individual threat level estimates did not change the pattern of group differences, which renders it unlikely that group differences in threat level learning account for group × threat level interactions. When the predator woke up, patients were caught and lost tokens more often (controls, 71.9%; patients, 95.6%; t = 3.90, p < 0.005), but because they collected many more tokens before the predator woke up, the reduction in overall earnings was less pronounced and did not reach significance (controls, 3.1; patients, 2.1; t = −1.83, p = 0.08, two-tailed). Accounting for overall earnings did not change the pattern of group differences. Thus, under stringent analysis, we found no support for the idea that patients’ behavior is explained by deficits in spatial navigation, hyperactivity, explicit memory for threat levels, or global strategic planning.

We provide evidence that approach-avoidance conflict results in behavioral inhibition and passive avoidance behaviors in humans, as in animal models of anxiety. Threat level in this model engages anterior hippocampus, while lesions to this structure attenuate these behaviors, possibly by impairing threat monitoring. One important difference between our human test and rodent analogs is that in rodent test beds, threat is not usually varied across levels. Our inclusion of varying threat levels allowed us to uncover a parametric relationship between passive avoidance behaviors and threat. Future pharmacological experiments in rodents and humans might benefit from the type of manipulation we describe and could address whether anxiolytics impact on sensitivity to threat (where the absolute reduction in passive avoidance/behavioral inhibition depends on threat level), or via a global reduction in behaviors associated with approach-avoidance conflict which does not depend on threat level. We note that one of the few rodent studies investigating this question has observed both patterns depending on the drug [19]. Our results suggest that both patterns occur simultaneously after hippocampus lesions.

We cannot fully exclude that subtle (histological) amygdala pathology might contribute to our results, particularly in light of a subthreshold amygdala activation in our fMRI experiment. Nevertheless, it is the anterior hippocampus that shows the most robust BOLD signal and the most pronounced lesion effect, both in our patient sample and in previous TLE samples [20]. All TLE patients were on antiepileptic medication which might—despite a lack of positive evidence in controlled human studies [21, 22]—exert anxiolytic effects, and we cannot fully exclude that this as a potential (albeit unlikely) explanation for the observed patient/control group differences. Our task sought to model a heterogeneous set of rodent tasks, though we recognize that doing this is complicated, not least by likely species-specific behavioral tendencies. Despite heterogeneity in these rodent tasks, lesions of the ventral hippocampus result in decreased behavioral inhibition and passive avoidance in all tasks. Future work will explore variants of our task that more closely model specific aspects of particular rodent paradigms.

This study helps answer what kind of human anxiety behavior is in fact modeled by approach-avoidance conflict. Human anxiety paradigms often capitalise on social threat [23, 24], explicit anticipation of pain [25], or unpredictable threat induced explicitly or by context conditioning [26]. The latter is suggested to involve the amygdala and bed nucleus of the stria terminalis [26]. It remains to be shown which, if any, of these different anxiety models captures the core features of clinical anxiety states, something rodent tests were originally developed to reflect. Indeed, our data, by putting the hippocampus center stage for approach-avoidance behavior, implicitly hint at novel conceptualisations of anxiety, including a possible link to systems mediating spatial behavior. Since anxiety behavior and spatial behavior may dissociate within the hippocampus [2], an intriguing question that awaits clarification is why core symptoms of clinical anxiety manifest phenomenologically with strong spatial referents, as exemplified in agoraphobia.

## Experimental Procedures

### General

Healthy volunteers took part in experiments 1–4, and patients with hippocampus sclerosis in experiment 4. Experiments 1 and 2 investigated behavior under approach-avoidance conflict with two slightly different realizations of threat levels. Experiment 3 involved an fMRI scanning session after initial training, during which we investigated BOLD responses to threat level. Experiment 4 examined the behavioral impact of hippocampus lesions. All experiments were approved by the respective local ethics committees.

Participants for experiments 1–3 were recruited from the general population in London (experiment 1: 12 male, 12 female, 23.3 ± 4.69 years; experiment 2: 12 male, 13 female, 23.1 ± 3.73 years; experiment 3: ten male, nine female, 23.1 ± 4.67 years); all samples were independent. Patients for experiment 4 (four male, three female, 46.6 ± 5.29 years) were recruited from the University Hospital of Barcelona epilepsy outpatient clinic. Unaffected relatives and friends of the patients were recruited as control participants (six male, six female, 43.4 ± 12.09 years).

Threat levels were denoted by frame color (Figures 1A–1D) that indicated distinct probabilities (0.2, 0.5, 0.8) of catching the human player who would then loose all tokens from the epoch; these probabilities were learned during the game and not explicitly signaled. We dynamically adjusted predator speed in experiment 1 and varied wake-up predator probabilities in experiments 2–4. Starting corner and epoch duration were varied randomly (see the Supplemental Experimental Procedures for technical implementation). Participants’ payment depended on performance in a randomly drawn sample of ten epochs. In experiments 1 and 4, we realized 120 (experiment 1) or 240 (experiment 4) epochs during which subjects learned the threat levels. To ensure stable strategies for experiments 2 and 3, we had participants explore the threat levels thoroughly (600 or 480 training epochs, respectively) and tested them on the next day (240 or 192 test epochs, respectively). Generally, behavioral indices asymptoted after some 20–40 epochs per condition, across the group.

The maximum speed of the human player was ten grid blocks per second. We averaged positions over 1 s bins and calculated dependent measures at this time resolution. The variable epoch duration implied that data from more epochs was used to estimate behavioral indices at earlier than at later time bins. Nevertheless, variance of dependent measures did not systematically increase with time, suggesting that there were sufficiently many data points for later time bins. Statistical analysis was carried out in R using a full multistratum repeated-measures ANOVA model with Greenhouse-Geisser correction for degrees of freedom. Bonferroni correction was applied for seven dependent measures per experiment. We report F tests or two-tailed t tests.

### Functional Magnetic Resonance Imaging

In experiment 3, images were acquired on a 3 T head scanner (Allegra, Siemens Medical Systems) with a single-channel head coil. Anatomical images of each subject’s brain were collected using an in-house multiecho 3D fast angle low shot (FLASH) sequence for mapping proton density, T1, and magnetization transfer, from which T1 weighted images were generated (voxel size, 1 × 1 × 1.5 mm) [27]. Field maps were acquired with the standard manufacturer’s double echo gradient echo field map sequence (echo time [TE], 10.0 and 12.46 ms; repetition time [TR], 1,020 ms; matrix size, 64 × 64), using 64 slices covering the whole head (voxel size, 3 × 3 × 3 mm). For functional images, we used BOLD signal-sensitive T2^∗^-weighted transverse single-shot gradient echo echoplanar imaging (EPI) (TE, 30 ms; effective TR, 2,880 ms; bandwidth in PE direction, 47.3 Hz/pixel; flip angle, 90°) after performing the manufacturer’s standard automatic 3D-shim procedure. Each volume contained 48 slices of 2 mm thickness (1 mm gap between slices; matrix size, 64 × 72; field of view, 192 × 216 mm). BOLD sensitivity losses in the hippocampus due to susceptibility artifacts were minimized by application of a z-shim gradient moment of 0.6 mT/m^∗^ms, a slice tilt of −45°, and a positive PE gradient polarity. In each of four scanning sessions, 200–300 functional whole-brain volumes were acquired. The first five volumes of each session were discarded to obtain steady-state longitudinal magnetization.

Image analysis was carried out using statistical parametric mapping (SPM8). EPI images were generated offline from the complex k-space raw data using a generalized reconstruction method based on the measured EPI k-space trajectory to minimize ghosting and were corrected for serial slice acquisition [28–30]. Images were corrected for geometric distortions caused by susceptibility-induced field inhomogeneities. These were estimated using the SPM8 FieldMap toolbox. The echoplanar images were then realigned and unwarped, a procedure that includes the measured static distortions in the estimation of motion-related distortion changes. Images were then coregistered to the individual’s T1 weighted image using a rigid body transformation and were normalized to the Montreal Neurological Institute (MNI) T1 reference brain template (resampled voxel size, 1.5 × 1.5 × 1.5 mm) using the DARTEL toolbox [31]. Normalized images were smoothed with an isotropic 8 mm full width at half-maximum Gaussian kernel. The time series in each voxel were highpass filtered at 1/128 Hz.

Threat level was entered into a GLM as boxcar function over the entire foraging phase. Each movement was modeled with a stick function and parametrically modulated by game phase (foraging versus chase), x position, y position, number of collected tokens, and instantaneous speed. Each change of direction was modeled as a separate event. Further variables were entered as continuous variables into the design matrix: x and y position, distance from predator, and number of collected tokens. Further, the design matrix included a boxcar function for the duration of each chase phase, a stick function for each of foraging start, foraging end without predator waking up, predator wake up, being caught by predator, end of chase phase, block start, block end, token collection, and automatic token update. All regressors were convolved with a hemodynamic response function. Further regressors were estimated movement parameters. From the within-subject model, we computed a linear contrast of threat level. As standard for a regression analysis, SPM8 reports only variance not explained by other regressors, such that all other predictors are effectively partialled out of the analysis. We then performed a second-level one-sample t test on contrast images from all participants (df = 18). Results were corrected for family-wise error using the SPM random field theory based approach [32].

### Lesion Study

For experiment 4, diagnosis of TLE+HS was established according to clinical electroencephalography (EEG) and MRI data. All patients underwent neurological and neuropsychological examination (Table S3) and continuous video EEG monitoring. Patients were included in the study when clinical data and MRI and EEG findings suggested mesial TLE. All patients had (1) seizures with typical temporal lobe features that were not controlled with antiepileptic drugs and (2) abnormally increased FLAIR signal on the left hippocampus, right hippocampus, or both. Antiepileptic drugs treatment is reported in Table S3.

A diagnostic MRI scan (Figure S2) was performed in all patients at the Bellvitge University Hospital with a 1.5 T unit (Philips Medical Systems, Best) in three orthogonal planes, including T1w (slice thickness = 1.1 mm; no gap; number of slices = 150; TR = 25 ms; TE = 4.60 ms; matrix = 320 × 320; field of view [FOV] = 240 mm; voxel size = 0.75 × 0.75 × 1.1 mm), T2w (slice thickness = 2 mm; no gap; number of slices = 80; TR = 2,500 ms; TE = 12 ms; matrix = 256 × 256; FOV = 230 mm; voxel size = 0.89 × 0.89 × 2 mm), and FLAIR (slice thickness = 5.2 mm; no gap; number of slices = 19; TR = 7,295 ms; TE = 12 ms; matrix = 256 × 256; FOV = 230 mm; voxel size = 0.89 × 0.89 × 5.2 mm) images. MRI scans were assessed by two experienced neurologists and one neuroradiologist who found no structural abnormalities besides hippocampal sclerosis unilaterally (TLE+UHS) or bilaterally (TLE+BHS). Volumetric comparison between groups relied on additional high-resolution T1-weighted images (Figure S2 and Table S4).

## Figures and Tables

**Table 1:** Inference Statistics on Defensive Behaviors

	Distance from Threat	Distance from Walls	Presence in Safe Place	Presence in Safe Quadrant	Presence in Threat Quadrant	Tokens per Second	Speed When on Grid
**Experiment 1 (n = 24)**							

Threat level: overall	19.15^∗∗∗^	22.69^∗∗∗∗^	16.87^∗∗∗^	17.31^∗∗∗^	4.92	18.83^∗∗∗^	9.47^∗^
Threat level: linear	5.87^∗∗∗∗^	−6.50^∗∗∗∗^	5.71^∗∗∗∗^	5.58^∗∗∗∗^	−2.47	−5.95^∗∗∗∗^	−4.34^∗∗∗^
Threat level: quadratic	−1.97	1.75	−1.08	−1.87	1.93	1.5	0.28
Task: overall	14.70^∗∗∗∗^	−0.76	6.44^∗∗∗∗^	10.78^∗∗∗∗^	−20.69^∗∗∗∗^	−3.67^∗∗^	2.55
Time: overall	277.23^∗∗∗∗^	85.94^∗∗∗∗^	41.87^∗∗∗∗^	118.61^∗∗∗∗^	707.86^∗∗∗∗^	71.18^∗∗∗∗^	211.51^∗∗∗∗^
Threat level: overall	0.46	0.88	0.01	2.11	0.17	0.38	5.51(^∗^)
Threat level × time: overall	924.30^∗∗∗∗^	6.57^∗∗∗∗^	142.96^∗∗∗∗^	254.44^∗∗∗∗^	878.87^∗∗∗∗^	3.41^∗∗∗^	46.65^∗∗∗∗^
Threat level × time: linear-linear	5.77^∗∗∗^	6.69^∗∗∗∗^	9.29^∗∗∗∗^	5.44^∗∗∗∗^	3.29^∗∗^	4.44^∗∗∗∗^	4.99^∗∗∗∗^
Threat level × time: quadratic-linear	7.17^∗∗^	−9.12^∗∗∗∗^	14.70^∗∗∗∗^	5.97^∗∗^	4.42^∗^	−7.12^∗∗∗∗^	−8.88^∗∗∗∗^
Task × time: overall	−4.20(^∗^)	5.96^∗∗∗^	−6.08^∗^	−3.80(^∗^)	0.04	4.85^∗∗∗^	0.09
Threat level × task × time: overall	0.91	1.02	1.09	1.05	2.27(^∗^)	0.66	1.15

**Experiment 2 (n = 25)**							

Threat level: overall	31.50^∗∗∗∗^	31.03^∗∗∗∗^	25.96^∗∗∗∗^	29.85^∗∗∗∗^	19.56^∗∗∗^	27.00^∗∗∗∗^	10.53^∗^
Threat level: linear	7.83^∗∗∗∗^	−7.81^∗∗∗∗^	7.17^∗∗∗∗^	7.63^∗∗∗∗^	−6.02^∗∗∗∗^	−7.30^∗∗∗∗^	−4.59^∗∗∗^
Threat level: quadratic	−1.28	1.01	−0.67	−1.21	1.71	0.85	−0.15
Task: overall	19.90^∗∗∗∗^	−1.94	7.79^∗∗∗∗^	14.32^∗∗∗∗^	−24.60^∗∗∗∗^	−5.34^∗∗∗^	1.23
Time: overall	441.74^∗∗∗∗^	135.44^∗∗∗∗^	73.47^∗∗∗∗^	251.44^∗∗∗∗^	849.40^∗∗∗∗^	149.30^∗∗∗∗^	146.94^∗∗∗∗^
Threat level: overall	0.38	1.28	0.04	0.78	0.01	0.28	2.63
Threat level × time: overall	1107.96^∗∗∗∗^	5.87^∗∗∗^	312.25^∗∗∗∗^	451.87^∗∗∗∗^	938.78^∗∗∗∗^	9.64^∗∗∗∗^	41.23^∗∗∗∗^
Threat level × time: linear-linear	16.50^∗∗∗∗^	14.91^∗∗∗∗^	19.28^∗∗∗∗^	14.69^∗∗∗∗^	3.76^∗∗^	12.82^∗∗∗∗^	7.89^∗∗∗∗^
Threat level × time: quadratic-linear	15.92^∗∗∗∗^	−15.15^∗∗∗∗^	20.42^∗∗∗∗^	12.24^∗∗∗∗^	−0.21	−16.02^∗∗∗∗^	−12.85^∗∗∗∗^
Task × time: overall	−6.09^∗^	4.67^∗^	−4.93(^∗^)	−5.98^∗∗^	1.7	4.00(^∗^)	−0.2
Threat level × task × time: overall	1.98	1.61	1.12	1.98	3.96^∗∗^	1.17	0.63

**Experiment 3 (n = 19)**							

Threat level: overall	31.50^∗∗∗∗^	31.03^∗∗∗∗^	25.96^∗∗∗∗^	29.85^∗∗∗∗^	19.56^∗∗∗^	27.00^∗∗∗∗^	10.53^∗^
Threat level: linear	7.83^∗∗∗∗^	−7.81^∗∗∗∗^	7.17^∗∗∗∗^	7.63^∗∗∗∗^	−6.02^∗∗∗∗^	−7.30^∗∗∗∗^	−4.59^∗∗∗^
Threat level: quadratic	−1.28	1.01	−0.67	−1.21	1.71	0.85	−0.15
Task: overall	19.90^∗∗∗∗^	−1.94	7.79^∗∗∗∗^	14.32^∗∗∗∗^	−24.60^∗∗∗∗^	−5.34^∗∗∗^	1.23
Time: overall	441.74^∗∗∗∗^	135.44^∗∗∗∗^	73.47^∗∗∗∗^	251.44^∗∗∗∗^	849.40^∗∗∗∗^	149.30^∗∗∗∗^	146.94^∗∗∗∗^
Threat level: overall	0.38	1.28	0.04	0.78	0.01	0.28	2.63
Threat level × time: overall	1107.96^∗∗∗∗^	5.87^∗∗∗^	312.25^∗∗∗∗^	451.87^∗∗∗∗^	938.78^∗∗∗∗^	9.64^∗∗∗∗^	41.23^∗∗∗∗^
Threat level × time: linear-linear	16.50^∗∗∗∗^	14.91^∗∗∗∗^	19.28^∗∗∗∗^	14.69^∗∗∗∗^	3.76^∗∗^	12.82^∗∗∗∗^	7.89^∗∗∗∗^
Threat level × time: quadratic-linear	15.92^∗∗∗∗^	−15.15^∗∗∗∗^	20.42^∗∗∗∗^	12.24^∗∗∗∗^	−0.21	−16.02^∗∗∗∗^	−12.85^∗∗∗∗^
Task × time: overall	−6.09^∗^	4.67^∗^	−4.93(^∗^)	−5.98^∗∗^	1.7	4.00(^∗^)	−0.2
Threat level × task × time: overall	1.98	1.61	1.12	1.98	3.96^∗∗^	1.17	0.63

**Experiment 4: Group Comparison (n = 7 TLE Patients, n = 12 Healthy Control Participants)**							

Group: overall	−2.98(^∗^)	1.49	−3.33^∗^	−3.54^∗^	2.26	−0.71	−0.81
Group × threat level: overall	2.21	3.48	0.7	2.82	0.28	3.83	1.12
Group × threat level: linear	−2.01	2.52	−1.07	−2.29	0.74	2.76(^∗^)	1.46
Group × task: overall	0.52	−0.06	−0.73	0.09	−0.19	1.63	2.45
Group × time: overall	7.82(^∗^)	5.96(^∗^)	2.84	6.96(^∗^)	2.83	5.87^∗^	2.74
Group × time: linear	−9.32^∗∗^	7.81^∗∗^	−4.93^∗^	−8.61^∗∗^	4.45^∗^	7.04^∗∗^	4.24^∗^
Group × threat level × time: overall	1.39	3.49^∗^	0.92	1.65	0.94	2.07	2
Group × threat level × time: linear	21.10^∗^	60.50^∗∗∗∗^	0.25	9.57	4.07	22.71^∗∗^	16.27^∗^

The table shows results from Experiments 1–3 on healthy participants, as well as comparison of seven patients with temporal-lobe epilepsy (TLE) with uni- or bilateral hippocampus sclerosis (HS) and 12 age- and gender-matched controls. We present F values (for overall condition effects) and signed t values (for polynomial contrasts and for the overall effect of task) from a 3 (condition) × 2 (task) × 17 (time) ANOVA (experiment 1), a 3 (condition) × 2 (task) × 15 (time) ANOVA (experiments 2 and 3), or a 2 (group) × 3 (condition) × 2 (task) × 15 (time) ANOVA (experiment 4). p values are corrected for nonsphericity according to Greenhouse-Geisser and are Bonferroni corrected for seven measures per experiment. Linear contrasts are coded as higher dependent values with higher levels of threat, and later time points, and quadratic contrasts as higher values for medium threat/time. (^∗^)p < 0.10, ^∗^p < 0.05, ^∗∗^p < 0.01, ^∗∗∗^p < 0.001, and ^∗∗∗∗^p < 0.0001.

## Supplemental Information

Document S1. Supplemental Experimental Procedures, Figures S1 and S2, and Tables S1–S4
