# Human Hippocampus Arbitrates Approach-Avoidance Conflict

**DOI:** 10.1016/j.cub.2014.05.051

**Published:** 2014-06-16

**Authors:** Dominik R. Bach, Marc Guitart-Masip, Pau A. Packard, Júlia Miró, Mercè Falip, Lluís Fuentemilla, Raymond J. Dolan

(Current Biology *24*, 541–547; March 3, 2014)

As a result of a production error, the version of Table 1 originally published in this article online and in print contained several incorrect labels. For each of experiments 1–3, the originally published table erroneously included two rows labeled “Threat level: overall,” the second of which should have read “Threat level × task: overall.” These erroneous labels have now been corrected in the article online. The journal and the authors apologize for any confusion resulting from these errors.

