# Supplementary material for: Human Hippocampus Arbitrates Approach-Avoidance Conflict
Source: Curr Biol. 2014 Mar 3;24(5):541–7. doi: 10.1016/j.cub.2014.01.046 (PMC3969259; doi:10.1016/j.cub.2014.01.046)
Supplement: Document S1. Supplemental Experimental Procedures, Figures S1 and S2, and Tables S1–S4 [file mmc1.pdf]

Current Biology, Volume *24*

Supplemental Information

Human Hippocampus Arbitrates

Approach-Avoidance Conflict

Dominik R. Bach, Marc Guitart-Masip, Pau A. Packard, Júlia Miró, Mercè Falip, Lluís Fuentemilla, and Raymond J. Dolan

Table S1, relating to figure 1: Subjective post-hoc estimates of the threat levels. Participants rated on a visual analogue scale (anchored 0%, 100%) how likely the predator would catch the participant if the human player was outside the safe place when it woke up (experiment 1), or how likely the predator would wake up (experiments 2-4). Experiment 4 entailed a final round on which participants could chose the colour they wanted to play by three pair-wise comparisons between the three predators. The table states the number of times that each predator was chosen. All data are given as mean ± standard deviation.

Experiment 4, probability ratings: Main effect of group $F(1, 17) < 1$ (n. s.); main effect of threat level $F(2, 34) = 7.8$ ($p < .005$); interaction group x threat level $F(2, 34) = 3.0$ ($p = .06$)

Experiment 4, preference ratings: Main effect of group $F(1, 17) = 1$ (n. s.); main effect of threat level $F(2, 34) = 5.7$ ($p < .01$); interaction group x threat level $F(2, 34) = 1.9$ (n.s.)

**True probability**	**20%**			**50%**			**80%**		
Experiment 1	41.6%	±	24.3%	62.3%	±	23.7%	81.6%	±	17.4%
Experiment 2									
after day 1	42.1%	±	24.9%	57.4%	±	18.2%	67.5%	±	21.3%
after day 2	39.0%	±	21.7%	54.8%	±	18.2%	68.7%	±	20.0%
Experiment 3									
after day 1	35.7%	±	24.8%	54.4%	±	20.4%	70.7%	±	19.9%
after day 2	22.4%	±	17.2%	58.0%	±	15.3%	72.7%	±	23.7%
Experiment 4									
control	32.0%	±	20.2%	51.4%	±	25.1%	76.7%	±	19.5%
patients	53.5%	±	13.1%	48.7%	±	24.5%	59.2%	±	23.0%
Experiment 4, Preference									
control	1.7	±	0.5	1.0	±	0.7	0.3	±	0.7
patients	1.0	±	1.0	1.3	±	1.0	0.7	±	0.5

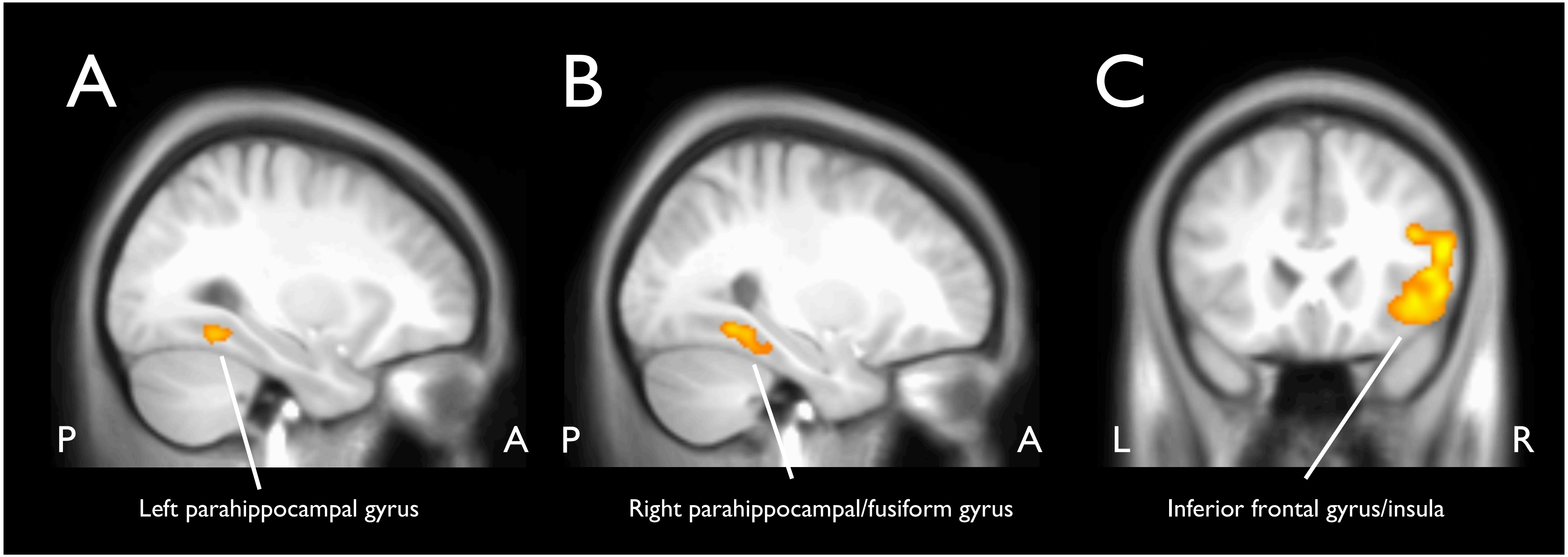

Figure S1, relating to figure 1: BOLD responses outside regions of interest, whole-brain corrected for family wise error at the cluster level, p<.05, and overlaid on a group average of T1 weighted images in MNI space. A: Sagittal view at x = -29, showing a cluster in the left parahippocampal gyrus (468 voxels, peak T = 5.24, peak coordinates -20/-54/-10). B: Sagittal view at x = 29, showing a cluster in the right fusiform/parahippocampal gyrus (940 voxels, peak T = 6.03, peak coordinates 44/-48/-24, 32/-48/-10, 35/-42/-21). C: Coronal view at y = 10, showing a cluster in the right inferior frontal gyrus/insula (3133 voxels, peak T = 6.59, peak coordinates 51/15/0, 52/19/15, 43/12/12).

Table S2, relating to figure 3: Within-group behavioural results from experiment 4. We present F-values (for overall condition effects) and signed t-values (for polynomial contrasts and for the overall effect of task) from a 3 (condition) × 2 (task) × 15 (time) ANOVA. P-values are corrected for non-sphericity according to Greenhouse-Geisser, and are Bonferroni-corrected for 7 measures per experiment. Linear contrasts are coded as higher dependent values with higher levels of threat, and later time points, and quadratic contrasts as higher values for medium threat/time. * p < .05; ** p < .01; *** p < .001; **** p < .0001

	Distance from threat	*Distance from walls*	*Presence in safe place*	*Presence in safe quadrant*	*Presence in predator quadrant*	*Tokens per second*	*Speed when on grid*
**Control participants (N = 12)**							
Threat level overall	5.89(*)	8.81*	1.59	6.86(*)	2.09	7.60*	0.59
Threat level linear	3.42*	-4.13**	1.6	3.67**	-2.04	-3.90**	-1.09
Threat level quadratic	-0.32	0.76	-0.78	-0.48	-0.04	0.04	-0.06
Task overall	7.52****	2.33	8.22****	7.47****	-6.95***	-0.94	1.24
Time overall	29.80***	13.24**	3.67	8.84*	98.58****	18.88****	17.50****
Threat level x task overall	0.18	0.53	0.2	0.02	1.07	0.1	0.77
Threat level x time overall	162.73****	2.36	20.31****	113.53****	98.73****	3.28*	10.75***
Threat level x time linear-linear	3.16	4.44***	1.26	3.84**	0.62	3.35**	1.69
Threat level x time quadratic-linear	7.94**	-9.40****	0.97	7.33***	-0.71	-6.91****	-4.47*
Task x time overall	-0.68	2.45	-1.7	-1.74	-0.68	0.77	2.36
Threat level x task x time overall	1.09	1.61	0.8	0.41	1.86	1.83	1.6
**TLE Patients (N = 7)**							
Threat level overall	0.39	0.38	0.28	0.59	0.43	0.2	0.66
Threat level linear	0.86	-0.16	-0.71	0.35	-0.78	-0.52	1
Threat level quadratic	0.22	-0.86	0.22	1.03	0.51	0.36	0.56
Task overall	8.58***	1.04	4.91*	6.95**	-9.08***	3.39	4.13*
Time overall	3.41	55.73****	3.87(*)	9.88**	41.96****	16.61****	10.77**
Threat level x task overall	0.48	1.5	0.93	0.2	0.15	0.68	1.65
Threat level x Threat level x time time overall	167.07****	0.83	23.68****	94.52****	159.24****	1.31	23.43****
Threat level x time linear-linear	0.6	1.05	0.65	0.64	1.37	0.86	1.5
Threat level x time quadratic-linear	1.01	2.27	1.34	1.49	-3.14(*)	0.51	1.88
Task x time overall	-0.37	-0.26	0.82	0.05	1.86	0.9	0.73
Threat level x task x time overall	0.81	0.86	0.71	0.37	0.8	0.53	1.04

Neuropsychological results for experiment 4

Neuropsychological data for all participants are summarized in Table S3. All participants (patients and controls) completed the Logical memory I (immediate verbal memory) and II (delayed verbal memory), the Visual reproduction I (immediate visual memory) and II (delayed visual memory), and the Digits Span, subtests of the Wechsler Memory Scale III [S1], the Vocabulary (IQ estimation) subtest of the Wechsler Adult Intelligence Scale [S2].

Given the large number of comparisons, threshold of statistical significance ($p < 0.05$) was adjusted by a Bonferroni correction that took into account the number of contrasts made (i.e., $p = (0.05/7) = 0.007$). Although group differences in the immediate verbal memory subtest failed to pass the corrected significance threshold ($F(1,18) = 5.78$, $p = 0.03$), we found significant differences between groups for the delayed verbal memory subtest ($F(1,18) = 14.48$, $p = 0.001$). Furthermore, group differences in the immediate visual memory subtest ($F(1,18) = 6.94$, $p = 0.02$) and delayed visual memory subtest ($F(1,18) = 8.19$, $p = 0.01$) did not pass the corrected threshold. Finally, the ANOVA contrasting the IQ estimation measure (vocabulary subtest) showed no differences between groups ($F(1,18) = 2.25$, $p = 0.15$).

Table S3, relating to figure 3. Demographic, medical, and neuropsychological data for patients with TLE with bilateral hippocampal sclerosis (BHS) and unilateral hippocampal sclerosis (UHS), and for control individuals included in the study. Age, years of education (Educ.), age at epilepsy onset (onset), seizure type (type): complex partial seizures (cps). Antiepileptic Drugs (AEDs): Valproate (VPA), Lamotrigine (LMT), Phenobarbital (PB), Phenytoine (PHT), Zonisamide (ZNS), Eslicarbazepine (ESL), Levetiracetam (LEV), Topiramate (TPM), Lacosamide (LCM), Oxcarbazepine (OXC), Carbamazepine (CBZ), Milligrams per day (MG/D). CPS: Complex partial seizure. The neuropsychological measures are LMI (Logical Memory I), LMII (Logical Memory II), VRI (Visual Reproduction I), VRII (Visual Reproduction II), Dig_span (Digits Span), Voc (Vocabulari) and IQ (estimated from Vocabulary subtest).

* This patient was under medication with Phenobarbital which binds to the benzodiazepin receptor and might have an anxiolytic effect in animals [S3]. Excluding this patient from the analysis did not affect the pattern of group differences.

Patient	Group	Age	Gender	Educ.	onset	type	Freq	Medication (AEDs)	LMI	LMII	VRI	VRII	Dig_span	Voc	IQ
AVC	UHS -R	46	F	16	8	Cps	5/month	VPA 1500MG/D, OXC 1200MG/D	2	1	7	5	6	6	80
ERL	UHS -R	50	M	16	1	Cps	2/week	CBZ 600MG/D, LEV 2000MG/D	5	4	7	4	8	11	105
PLO	UHS -L	37	M	12	14	Cps	3/month	LEV 1000MG/D, VPA 500MG/D, LMT 200MG/D	11	9	10	9	12	8	90
LGP	BHS	43	F	12	12	Cps	3/week	CBZ 1600MG/D, PB 150MG/D *	4	3	5	6	4	8	90
VC	BHS	54	F	14	45	Cps	3-5/week	VPA 2000MG/D, LMT 400MG/D	6	4	5	4	7	9	95
JRG	UHS -L	54	M	9	2	Cps	3/15days	LEV 3000MG/D, OXC 2100MG/D, LCM 600MG/D	6	4	5	4	7	9	95
PRG	UHS -R	50	M	9	8	Cps	5/month	CBZ 600MG/D, LEV 4000MG/D, ZNS 200MG/D	6	3	5	7	10	12	110
MP	Control	41	F	6					5	5	5	3	11	10	100
DDA	Control	32	M	22					10	8	11	9	13	11	105
PCV	Control	25	F	14					10	10	12	10	7	8	90
JB	Control	43	F	12					16	16	9	10	16	14	120
FLM	Control	51	M	6					11	14	7	7	11	11	105
AS	Control	21	M	14					8	9	14	11	5	13	115
NB	Control	42	F	9					7	8	17	10	11	12	110
CA	Control	53	F	6					6	8	6	8	11	10	100
PSY	Control	55	M	6					11	12	9	11	8	8	90
IG	Control	35	F	12					10	11	10	7	7	11	105
JFR	Control	52	M	6					5	5	14	7	7	9	95
VMD	Control	57	M	6					11	8	8	9	8	8	90

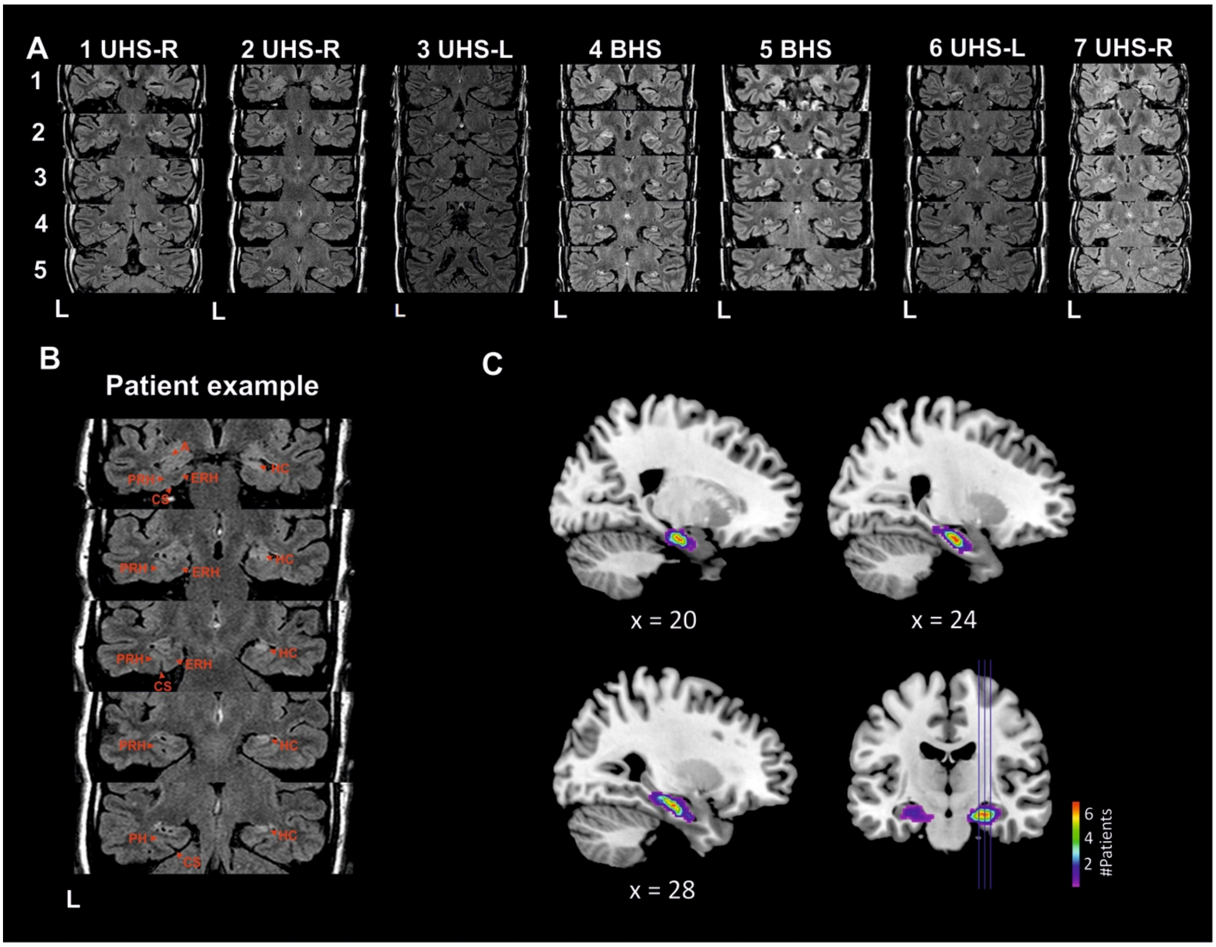

Figure S2, relating to figure 3. (A) Series of FLAIR coronal images for each of the 7 TLE+HS patients and one representative control. The sections proceed in 5 mm intervals from the perirhinal, entorhinal cortex and hippocampus in the top sections caudally through the hippocampus and the parahippocampal cortex surrounding the collateral sulcus in the bottom section. The top section shows the collateral sulcus and surrounding perirhinal and entorhinal cortices with anterior hippocampus. Also at this level the amygdala (A) can be seen. The second and third sections show the hippocampus and the perirhinal and enthorinal cortices. Extensive hippocampal damage is evident at these levels in all patients with reduced volume of the right hippocampus in patients 1, 2, 7; left hippocampus in patients 3 and 6 and bilateral hippocampus in 4 and 5. Temporal lobe atrophy and dilatation of temporal horn accompany the ipsilateral hippocampal atrophy. The fourth section shows perirhinal cortex on the lateral bank of the colateral sulcus, near the perirhinal/parahippocampal cortex border. The fifth section shows the hippocampus and the colateral suclus, surrounded by parahippocampal cortex. (B) As described by [S4], the collateral sulcus (CS) is the most important structure for the identification of medial temporal lobe cortices. At its most rostral extent, the collateral sulcus is surrounded entirely by perirhinal cortex (PRH).

Caudally, entorhinal cortex (EC) extends from the midpoint of the medial bank of the collateral sulcus to the subiculum, whereas perirhinal cortex extends laterally from the midpoint of the medial bank of the collateral sulcus to the inferotemporal cortex. Two millimeters caudal to the disappearance of the gyrus intralimbicus of the hippocampus (HC), the collateral sulcus is surrounded by parahippocampal cortex (PH). (C) The extent of hippocampal sclerosis for each patient in the TLE+HS group was defined on the FLAIR image via manually depicting areas of hyperintensity. These lesioned areas were defined by an experienced neuroradiologist using the MRIcron software package [S5] (www.cabiatl.com/mricro/mricron/index.html) and transformed into binary masks of sclerotic tissue. The volume for each sclerotic mask was extracted using SPM8. To compute an anatomical overlap of the sclerotic regions of the hippocampus each FLAIR image for the TLE patient was segmented and normalised using Unified Segmentation [S6] with medium regularisation and cost function masking [S7-9] and the previously defined sclerotic binary masks. Each sclerotic binary mask was then normalized to the MNI template by means of the normalization parameters derived from the previous step. Finally, all binary masks were flipped to the right hemisphere and added together to compute the anatomical overlap. Note that coloured area in the left hippocampus belongs to the sclerotic region of the two TLE+BHS patients of the group, which was also located in the anterior portion of the hippocampus.

Table S4, relating to figure 3. Bilateral hippocampus, amygdala and parahippocampus volume for controls and TLE+HS patients. Mean (SEM) volume in ml for bilateral hippocampus, amygdala, parahippocampal gyrus, and total intracranial volume (TIV), compared between the groups with an independent sample t-test. Volumetric analysis shows that only the hippocampal volume for the TLE group is significantly lower than that for the matched control group.

	Hippocampus	Amygdala	Parahip.	TIV
**TLE patients**	3.17 (0.41)	1.88 (0.61)	4.24 (0.65)	1434.3 (92.1)
**Controls**	4.32 (0.50)	1.68 (0.32)	4.57 (0.41)	1528.6 (179.8)
	**t(12)=4.35**	t(12)=1.10	t(12)=1.14	t(12)=1.23
	**p<0.001**	p=0.29	p=0.27	p=0.24

Supplemental Experimental Procedures

(1) Task setup

A 24 × 16 grid in landscape orientation was presented almost full-screen on a standard computer monitor, with white quadratic grid blocks, separated by black gridlines and surrounded by a white inner frame. An outer frame indicated the colour of the sleeping predator. When the predator woke up, the inner frame turned to dark grey, and the outer frame to red. The human player was represented by a green triangle, and the sleeping predator by a grey circle, assuming its true colour once it woke up. The association of predator colours (blue, orange, purple) with threat levels was randomly balanced across participants. Ten tokens, represented by yellow rhombi, were randomly distributed on the grid. Every 2 seconds one of the tokens randomly changed its position, and every collected token was immediately replaced by a new token in random position on the grid. Head-screen distance was at the participant's discretion expect for experiment 3.

The human player was controlled with the cursor keys on a standard computer keyboard, and with a 4-way keypad for the imaging experiment 3. No diagonal movements were possible for both human player and predator. Continuous key presses moved the human player at its maximum speed of 10 blocks per second. If the human player moved over a token, this token disappeared from the grid and was shown in a row above the grid.

In experiment 1, the predator would wake up on every epoch, and we dynamically adjusted its speed such that the target catch probability was met over a moving window of 10 epochs of the same type on which the player was outside the safe place when the predator woke up. This implied that participant were able to indirectly alter the predator's speed which might induce dynamic change of strategy over time. In experiments 2-4, all three predators had the same speed of 40 grid blocks per second, and level of threat was defined as probability of waking up. When the predator did not wake up, an epoch lasted 15 seconds and then terminated. Otherwise, an epoch lasted until 5 seconds after wake up, independent on whether or not the human player was caught. This design implied that the hazard rate was increasing over time for experiments 2-4. If the predator caught the human player, the human player would disappear, the predator would turn red, all tokens would disappear, and the predator

would stay on the grid block where the subject was caught until the 5 seconds of the catch phase were over.

Task type (active, passive), threat level, starting corner, and epoch duration were randomly determined in advance, and balanced across epochs. In experiment 1 we realised 5 durations (3 to 17 seconds in 3.5 second steps), and all combinations with starting corner, task, and threat, thereby creating 120 unique epochs. In experiment 2, there were 120 unique epochs (duration 3 to 15 seconds in 3 second steps), repeated 5 times for training on day 1 (600 epochs), and twice on day 2 (240 epochs). For experiment 3, we created 480 unique training epochs with 20 possible durations between 3 and 15 seconds, and in the MRI scanner, 198 unique testing epochs with 8 possible durations between 3 and 15 seconds. In experiment 4, there were 192 unique epochs (duration 3 to 15 seconds in 1.5 second steps) and an additional 48 unique epochs (duration 3 or 4.5 seconds). To render the task manageable for patients in experiment 4, we introduced procedural modifications such that epochs of the game were separated by slightly longer breaks, and participants had the chance to see the grid layout before an epoch started. This did not alter the pattern of behaviour in the control group (table S2).

(2) Supporting behavioural analyses for experiment 1-3

Because the behavioural measures might appear closely related, we calculated, for each experiment separately, a principal component analysis for seven time-dependent variables, in order to probe whether they could be summarised. We used data averaged across epochs for all participants, conditions, and timepoints. Scree plots revealed a multidimensional structure of these measures. The first two components explained 25-40% of the variance, and the next 4 components around 5-15% for all three experiments, with no clear cutoff. Factor loadings were very similar across experiments. This possibly maps onto complex behavioural strategies that are dynamically adapted over intra-epoch time, or between threat levels and tasks. The multi-dimensional structure mandated that we analysed the dependent measures individually, instead of collapsing them into summary scores, while correcting for multiple comparisons.

(3) Supporting fMRI analyses for experiment 3

In order to estimate mean BOLD signal per epoch, we constructed a model in which each epoch was represented by a regressor containing one boxcar function, convolved with a

hemodynamic response function. Additional control variables were the same as in the main analysis. We extracted mean BOLD signal from the activated cluster in our region of interest with standard SPM8 functions. Despite the lower precision of epoch-by-epoch parameter estimates, estimated BOLD signal in this cluster was significantly related to threat level, as expected from the main analysis. We then computed several epoch-summary measures, meant to index defensive behaviour across the entire epoch: relative duration of presence in safe place, average distance from threat, distance travelled. All these measures were, on an epoch-by-epoch basis, significantly related to threat level ($p < .01$ in a threat × task ANOVA). There was no association of these measures with estimated BOLD signal over and above the influence of threat level. We then constructed an fMRI model uncorrected for spatial variables. Over and above the influence of threat level, RoI signal was related to less presence in safe place and more distance travelled. This means that on an epoch-by-epoch basis, uncorrected BOLD signal is stronger when threat level is higher, but less strong when behaviour is more defensive (i. e. reduction in spatial navigation). This suggests that the observed BOLD responses in the main analysis were truly driven by threat level and not by specific defensive behaviours.

In order to explore the impact of between-subject differences in threat probability estimates, two supporting analyses were conducted. First, we regressed estimates of mean of subjective threat level, and its linear change (difference between level 1 and level 3) onto estimates of linear threat level relation with BOLD signal. No significant results emerged, even at an uncorrected significance threshold. Secondly, we extracted the mean BOLD signal from the activated cluster in our region of interest for the three threat levels. We then related mean and linear change of BOLD signal to mean and linear change of subjective probability estimates. Mean BOLD signal was related to mean subjective estimate of threat level ($t(17)=4.4$; $p<.005$, Bonferonni-corrected for 4 comparisons). Note that the cluster defining contrast (linear effect of threat level) is independent from the tested contrast (mean subjective probability estimates).

(4) Supporting analyses for experiment 4

(a) Tokens per second, speed: these variables were available on a moment-by-moment basis and were simply partialled out of all other dependent measures for the supporting analysis.

(b) Subjective threat level estimates, average token earnings: these control variables were available for each individual and threat level, but not for each time point. In a first step, to control the main effect of group and group × threat level interaction, they were regressed out of the dependent variables. These variables might however also account for a lack of intra-epoch behavioural adaptation in patients with hippocampal lesions. Hence, in a second step we controlled for interactions with time, and assumed a model for the behavioural variable *Y* at time *t*, epoch number *n*, threat level *i*, individual *j*

$$Y_{ij}(n,t) = \left(p_{ij} + s_{ij}\right)t + R_{nt}$$

according to which, for each individual *i* and threat level *j*, the linear change in the dependent variable *Y* over time is governed by a component s_{ii} which depends on the individual value of the control variable, and a residual component p_{ij}. The component s_{ii} was estimated by regressing, for each individual and threat level, the linear change in the dependent measure over time, averaged across epochs, onto the control variable. A corrected behavioural measure was then calculated as

$$Y'_{ij}(n,t) = Y_{ij}(n,t) - s_{ij}t$$

(4) Volumetric analysis for experiment 4

Additional high-resolution whole-brain MRI structural T1-weighted images were acquired for all TLE patients and seven out of the twelve control individuals (slice thickness = 1 mm; no gap; number of slices = 240; TR = 2300 ms; TE = 3 ms; matrix = 256 x 256; FOV = 244 mm; voxel size = 1x1x1 mm) was acquired using a 3.0 Tesla Siemens Trio MRI system at the Hospital Clinic of Barcelona.

Structural T1 images were processed using the FreeSurfer (http://surfer.nmr.mgh.harvard.edu, v5.2.0) pipeline, which includes the removal of non-brain tissue [S10], automated Talairach transformation, intensity normalisation [S11], segmentation of the subcortical white matter and deep grey matter [S12, 13], tessellation of the grey matter/white matter boundary, automated topology correction [S14, 15], and surface deformation to detect grey/white matter and grey matter/cerebrospinal fluid (CSF) boundaries [S16]. A deformable procedure was applied to parcellate the cerebral cortex into different regions according to gyral and sulcal structure information [S17].

The FreeSurfer pipeline in high-resolution T1-weighted images was used to extract the volume of the spared and the intact portion of the lesioned hippocampus in TLE patients and to identify the volume of the hippocampus in the control individual. Volume for the amygdale and parahippocampus girus was also extracted. Hippocampal, amygdala and parahippocampal volumes were corrected for Total Intracranial Volume (TIV) [S18, 19]. First, a linear regression with the extracted volumes as the dependent variable and TIV as the independent variable was performed. Using the unstandarised beta-weights of this correlation, the volumes were corrected as follows:

$$\mathbf{V_{ic}} = \mathbf{V_i} + (\mathbf{Mean_{TIV}} - \mathbf{TIV_i}\ ) * \mathbf{Beta}$$

where $\mathbf{V_{ic}}$ is the corrected volume for a particular structure and subject, $\mathbf{V_i}$ is the raw volume as calculated by Freesurfer, $\mathbf{Mean_{TIV}}$ is the mean TIV for a particular group, $\mathbf{TIV_i}$ is the specific TIV of the subject and **Beta** is the beta-weight calculated for this subject during the linear regression.

Using the corrected volumes, two-sample t-tests were performed between the TLE patients and controls. The results are summarised in Supplemental Table S7.

Supplemental references

S1. Wechsler, D. (2004). WMS-III. Escala de memoria de Wechsler-III, (Madrid: TEA).

S2. Wechsler, D. (1999). WAIS III Escala de Inteligencia de Wechsler para Adultos – III, (Madrid: TEA Ediciones).

S3. Gray, J.A., and McNaughton, N. (2000). The neuropsychology of anxiety: An enquiry into the functions of the septohippocampal system, Volume 2, (Oxford, UK: Oxford University Press).

S4. Insausti, R., Juottonen, K., Soininen, H., Insausti, A.M., Partanen, K., Vainio, P., Laakso, M.P., and Pitkanen, A. (1998). MR volumetric analysis of the human entorhinal, perirhinal, and temporopolar cortices. AJNR Am J Neuroradiol *19*, 659-671.

S5. Rorden, C., and Brett, M. (2000). Stereotaxic display of brain lesions. Behav Neurol *12*, 191-200.

S6. Ashburner, J., and Friston, K.J. (2005). Unified segmentation. Neuroimage *26*, 839-851.

S7. Andersen, S.M., Rapcsak, S.Z., and Beeson, P.M. (2010). Cost function masking during normalization of brains with focal lesions: still a necessity? Neuroimage *53*, 78-84.

S8. Brett, M., Leff, A.P., Rorden, C., and Ashburner, J. (2001). Spatial normalization of brain images with focal lesions using cost function masking. Neuroimage *14*, 486-500.

S9. Ripolles, P., Marco-Pallares, J., de Diego-Balaguer, R., Miro, J., Falip, M., Juncadella, M., Rubio, F., and Rodriguez-Fornells, A. (2012). Analysis of automated methods for spatial normalization of lesioned brains. Neuroimage *60*, 1296-1306.

S10. Segonne, F., Dale, A.M., Busa, E., Glessner, M., Salat, D., Hahn, H.K., and Fischl, B. (2004). A hybrid approach to the skull stripping problem in MRI. Neuroimage *22*, 1060-1075.

S11. Sled, J.G., Zijdenbos, A.P., and Evans, A.C. (1998). A nonparametric method for automatic correction of intensity nonuniformity in MRI data. IEEE Trans Med Imaging *17*, 87-97.

S12. Fischl, B., van der, K.A., Destrieux, C., Halgren, E., Segonne, F., Salat, D.H., Busa, E., Seidman, L.J., Goldstein, J., Kennedy, D., et al. (2004). Automatically parcellating the human cerebral cortex. Cereb Cortex *14*, 11-22.

S13. Fischl, B., Salat, D.H., Busa, E., Albert, M., Dieterich, M., Haselgrove, C., van der Kouwe, A., Killiany, R., Kennedy, D., Klaveness, S., et al. (2002). Whole brain segmentation: automated labeling of neuroanatomical structures in the human brain. Neuron *33*, 341-355.

S14. Fischl, B., Liu, A., and Dale, A.M. (2001). Automated manifold surgery: constructing geometrically accurate and topologically correct models of the human cerebral cortex. IEEE Trans Med Imaging *20*, 70-80.

S15. Segonne, F., Pacheco, J., and Fischl, B. (2007). Geometrically accurate topology-correction of cortical surfaces using nonseparating loops. IEEE Trans Med Imaging *26*, 518-529.

S16. Fischl, B., and Dale, A.M. (2000). Measuring the thickness of the human cerebral cortex from magnetic resonance images. Proc.Natl.Acad.Sci.U.S.A *97*, 11050-11055.

S17. Desikan, R.S., Segonne, F., Fischl, B., Quinn, B.T., Dickerson, B.C., Blacker, D., Buckner, R.L., Dale, A.M., Maguire, R.P., Hyman, B.T., et al. (2006). An automated labeling system for subdividing the human cerebral cortex on MRI scans into gyral based regions of interest. Neuroimage *31*, 968-980.

S18. Horner, A.J., Gadian, D.G., Fuentemilla, L., Jentschke, S., Vargha-Khadem, F., and Duzel, E. (2012). A rapid, hippocampus-dependent, item-memory signal that initiates context memory in humans. Curr Biol *22*, 2369-2374.

S19. Gadian, D.G., Calamante, F., Kirkham, F.J., Bynevelt, M., Johnson, C.L., Porter, D.A., Chong, W.K., Prengler, M., and Connelly, A. (2000). Diffusion and perfusion magnetic resonance imaging in childhood stroke. J Child Neurol *15*, 279-283.
